# Combination of Anti-VEGF and Laser Photocoagulation for Diabetic Macular Edema: A Review

**DOI:** 10.1155/2017/2407037

**Published:** 2017-02-27

**Authors:** Laura N. Distefano, Jose Garcia-Arumi, Vicente Martinez-Castillo, Anna Boixadera

**Affiliations:** Vall d'Hebron Hospital, Pg de la Vall d'Hebron 119, 08032 Barcelona, Spain

## Abstract

Diabetic macular edema (DME) is the most common cause of vision loss in diabetic patients. Thirty years ago, the Early Treatment Diabetic Retinopathy Study (ETDRS) demonstrated that focal/grid laser photocoagulation reduces moderate vision loss from DME by 50% or more; thus, macular photocoagulation became the gold standard treatment for DME. However, with the development of anti-VEGF drugs (bevacizumab, ranibizumab, and aflibercept), better outcomes were obtained in terms of visual acuity gain and decrease in macular thickness in some studies when antiangiogenic drugs were administered in monotherapy. Macular laser therapy may still play an important role as an adjuvant treatment because it is able to improve macular thickness outcomes and reduce the number of injections needed. Here, we review some of the clinical trials that have assessed the efficacy of macular laser treatment, either as part of the treatment protocol or as rescue therapy.

## 1. Introduction

More than four hundred million adults suffer from diabetes mellitus worldwide [1]. Ninety percent of diabetic patients will have some form of retinopathy twenty-five years following diagnosis [2]. Diabetic retinopathy causes blood-retinal barrier breakdown, leading to increased permeability and leakage from retinal capillaries. Fluid accumulates within the retinal layers, resulting in a thickened macula. Diabetic macular edema (DME) is the most common cause of vision loss in diabetic patients, with a prevalence that ranges from 19% to 65% [3]. This paper reviews the current role of laser for DME in the era of antiangiogenic therapy.

## 2. Diabetic Macular Edema

Involvement or threatening of the center of the macula was termed clinically significant macular edema (CSME) by the Early Treatment Diabetic Retinopathy Study (ETDRS) [4, 5]. Furthermore, in the ETDRS, edema was classified as focal or diffuse by the proportion of leakage that came from microaneurysms graded in fluorescein angiograms: more than 66% or less than 33%, respectively. Severity of macular ischemia was also graded according to the degree of capillary loss in the central and inner subfields [6]. Nowadays, however, DME is mainly classified by its central involvement in most of the main clinical trials that evaluate intravitreal therapy for DME.

## 3. Macular Laser Photocoagulation

### 3.1. Treatment Techniques

In the ETDRS, two macular laser treatment techniques were defined: focal and grid, both performed between 500 and 3000 microns from the fovea but not within 500 microns from the papillary border [7, 8]. *Focal* laser was applied for focal lesions that not only included microaneurysms but also included intraretinal microvascular abnormalities (IRMA) and small capillaries with focal leakage. *Grid* laser was performed in areas of macular thickening with diffuse leakage or capillary loss.

Although effective, laser burns close to fixation were described to enlarge over time, potentially affecting the fovea, with secondary central visual loss, central scotomas, and altered color vision [9]. To avoid these potential complications, a modification of the described ETDRS technique was developed, called the modified-ETDRS laser treatment (MLT) technique. In this modified protocol, a smaller spot of 50 *μ*m size and 50 to 100 milliseconds burn duration is performed. *Focal* laser aims to treat only leaking microaneurysms, causing a mild gray-white burn but not necessarily a darkening or whitening of the microaneurysm. *Grid* laser is applied to areas with diffuse leakage or nonperfusion, with barely visible, two-burn widths apart laser burns [10]. This is currently the preferred technique in daily practice, especially focal laser for treating small areas of thickening, threating but not affecting the fovea. MLT is, likewise, the protocol of choice in the studies we will describe below.

### 3.2. Mechanism of Action

*Focal* laser is thought to reduce the leakage from the microaneurysm by direct occlusion of the lumen [11]. On the other hand, although the exact mechanism of the effect of *grid* photocoagulation is not well understood, some plausible theories have been described [12]: (a) destruction of photoreceptors which have high oxygen demands, (b) increased oxygenation of the retina through the laser scar, (c) restoration of new RPE barrier by spreading in small lesions and by proliferation in larger lesions, (d) production of cytokines TGF beta and PEDF from the stimulated RPE, (e) decrease in area of abnormal leakage: reduction in the leaking capillary area, and (f) autoregulation with secondary decrease in retinal blood flow and consequent decreased edema [13].

## 4. Clinical Trials of Diabetic Macular Edema

Several prospective, randomized, controlled clinical trials have been conducted in the past years regarding the treatment of DME involving the center of the macula. We review here some of the ones that have compared macular laser treatment as part of the treatment protocol or as rescue therapy.

### 4.1. Early Treatment Diabetic Retinopathy Study (ETDRS)

In this classic study, patients with DME, visual acuity (VA) of 20/200 or better, and no proliferative retinopathy were randomized into early or deferred focal/grid photocoagulation [6]. DME eyes with CSME treated with laser had lower rates of visual loss compared with controls: 12 versus 24%, at 3 years. In diffuse edema, grid was of limited benefit: VA improved in 15%, worsened in 24%, and remained stable in 61% [4, 5]. Thus, the ETDRS demonstrated that focal/grid laser photocoagulation reduces moderate vision loss from DME by 50% or more, and from these results, it became the gold standard against which all new treatments have been compared to since then [4, 6].

### 4.2. Clinical Trials with Macular Laser as a Treatment Arm

#### 4.2.1. Intravitreal Triamcinolone Acetonide and Focal/Grid Photocoagulation for Diabetic Macular Edema (DRCR.net Protocol B Study)

One of the first drugs that was tested against laser treatment was intravitreal triamcinolone. In 2008, the first results from Diabetic Retinopathy Clinical Research Network (DRCR.net) Protocol B were published: eight hundred forty study eyes with VA of 20/40 to 20/320 were randomized to focal/grid photocoagulation, 1 mg or 4 mg intravitreal triamcinolone. Retreatment was administered for persistent or new edema at 4-month intervals. The results at 12 months showed no significant differences in mean VA gain among the three treatment arms. At the 2-year follow-up visit, mean VA change from baseline was clinically modest but statistically greater in the laser group (+1 ETDRS letter) than in the other 2 groups (−2 and −3 ETDRS letters; *p* = 0.02 and *p* = 0.002, resp.). This difference was observed even for those eyes that were pseudophakic at baseline and for those that were pseudophakic or had minimal or no cataract at the 2-year follow-up visit [14]. After three years of follow-up, VA letter score did not show any change in the triamcinolone groups but improved in 5 letters in the laser group [15]. Macular thickness in optical coherence tomography (OCT) decreased in a similar way. In terms of cataract formation, most eyes treated with 4 mg triamcinolone required surgery.

These results highlight the long-term effect of laser treatment monotherapy, as the outcomes in visual acuity and retinal thickness improved after three years of treatment.

#### 4.2.2. Bevacizumab or Laser Therapy (BOLT Study)

Two years later, the BOLT study evaluated intravitreal antiangiogenic 1.25 micrograms bevacizumab versus focal/grid photocoagulation in patients with persistent center-involving CSME and visual acuity of 20/40 to 20/320. Eighty eyes were randomized into receiving either bevacizumab alone (at 6-week intervals) or focal/grid alone (at 4-month intervals) [16]. At the 12-month follow-up visit, subjects treated with bevacizumab improved a median of 8 ETDRS letters, compared with the laser-treated eyes, which lost VA (−0.5, *p* = 0.0002), despite a median of 3 laser treatments being performed. Twelve percent of subjects treated with bevacizumab versus 5.3% treated with laser gained more than 15 letters. Consistently with VA results, central macular thickness (CMT) decreased 129 *μ*m in the bevacizumab group (*p* < 0.001) compared with 68 *μ*m in the laser group (*p* = 0.02).

At the 2-year follow-up visit, the bevacizumab arm maintained a median of +9 letters improvement, whereas laser-treated eyes showed better results than at 1-year visit (+2.5, *p* = 0.005). In terms of percentage of patients who gained 15 letters or more compared to baseline, the bevacizumab arm showed superiority compared to laser (32% and 4%, resp., *p* = 0.004). Mean reduction in central macular thickness was 146 *μ*m in the bevacizumab arm versus 118 *μ*m in the MLT arm [17]. Even though the follow-up was short, laser outcomes are slightly better during the second year compared with the first year.

#### 4.2.3. VEGF-Trap-Eye in Patients with Diabetic Macular Edema (DA VINCI Study)

This phase II clinical trial enrolled 221 diabetic patients, assigned to receive either 0.5 mg aflibercept every 4 weeks, 2 mg aflibercept every 4 weeks, 3 monthly injections of 2 mg aflibercept and then every 8 weeks, 3 monthly injections of 2 mg aflibercept and then on a PRN protocol, or macular laser photocoagulation alone.

All aflibercept groups gained VA ranging from a mean of +9.7 to +13.1 letters, while patients in the laser group only lost a mean of 1.3 letters at 1-year follow-up. Mean reductions in CMT in the 4 aflibercept groups ranged from −165 to −227 *μ*m compared with only −58 *μ*m in the laser group (*p* = 0.0066, aflibercept arms versus laser) [18].

#### 4.2.4. Intravitreal Aflibercept for Diabetic Macular Edema (VIVID/VISTA Studies)

More recently, 872 eyes were enrolled in two identical phase III trials that randomized them into receiving 2 mg aflibercept every 4 weeks, 5 monthly injections of 2 mg aflibercept and then every 8 weeks, or focal/grid laser. Mean VA improvement from baseline to week 100 was the lowest in the laser groups (+0.9 and +0.7) compared with that of both aflibercept arms (+11.5 and 11.4 for aflibercept every 8 weeks; +11.4 and +9.4 for aflibercept every 8 weeks) for VIVID and VISTA, respectively. The proportion of the eyes that gained 15 letters or more from baseline at week 100 was 38%, 33%, and 13% (*p* < 0.0001) in VISTA and 38%, 31%, and 12% (*p* < 0.0001) in VIVID [19].

DA VINCI and VIVID/VISTA show poor short-term results of laser alone in terms of visual acuity.

#### 4.2.5. Ranibizumab for Edema of the Macula in Diabetes (READ-2 Study)

The READ-2 study added the combination therapy as a treatment arm. For the first 6 months of the study, 126 patients with DME were randomized to receive 0.5 mg ranibizumab every two months, focal/grid laser photocoagulation at baseline and at month 3 if needed, or a combination of 0.5 mg of both at baseline and month 3. At month 6, the mean gain in BCVA was significantly greater for ranibizumab monotherapy (+7.24 letters, *p* = 0.01) compared with laser alone (−0.43 letters); combination therapy showed no differences when compared to the other two (+3.80 letters). Twenty-two percent, 0%, and 8% of the subjects improved 15 letters or more (*p* = 0.002). CMT was reduced as well by 50%, 33%, and 45% in groups 1, 2, and 3, respectively [20].

After month 6, all subjects were allowed to be treated with ranibizumab. Fewer injections were needed during the 18-month follow-up for the combination group (2.9) compared with ranibizumab alone (5.3) and laser alone (4.4) original groups. At 24 months, mean improvement in BCVA remained stable for ranibizumab-treated patients (+7.7) but was better for laser alone (+5.1) and combination therapy (+6.8). The percentage of patients who gained 15 letters or more of BCVA also improved for the laser and ranibizumab plus laser-treated eyes, compared to the 6-month results (24%, 18%, and 26% for ranibizumab, laser, and combination, resp.). In terms of mean CMT at the 24-month visit, combination therapy showed the better results: 258 microns, compared with 340 *μ*m achieved with ranibizumab and 286 *μ*m with laser alone [21].

At the 36-month visit, mean improvement from the baseline BCVA was greater compared to the 24-month results in the ranibizumab group (+10.3 letters). Laser (+1.4 letters) and combination (+8.9 letters) groups showed more stable results when compared with the 2-year results. However, CMT showed greater reduction with combination therapy (−243 *μ*m) than with laser alone (−193 *μ*m) or ranibizumab alone (−132 *μ*m). The mean number of ranibizumab injections was greater in the ranibizumab arm compared with the laser arm (5.4 versus 2.3 injections, *p* = 0.008) but not compared with the ranibizumab plus laser arm (3.3, *p* = 0.11) [22].

In brief, 3-year outcomes of combination therapy showed the greatest CMT reduction, greater VA change than laser but no ranibizumab, and fewer injections needed than ranibizumab monotherapy.

#### 4.2.6. Ranibizumab Monotherapy or Combined with Laser versus Laser Monotherapy for Diabetic Macular Edema (RESTORE Study)

Similar to the READ-2 study, combination therapy was included in this protocol: 345 diabetic patients with visual impairment due to DME were randomized to 0.5 mg ranibizumab (group 1), 0.5 mg ranibizumab plus laser (group 2), or sham injections plus laser (group 3). Ranibizumab was given monthly for the first 3 months then pro re nata (PRN); laser was given at baseline then PRN. Mean average change in BCVA letter score from baseline to month 1 through 12 was +6.1 and +5.9 versus +0.8 for groups 1, 2, and 3 (both *p* < 0.0001). No differences were found when comparing focal and diffuse types of edema. At month 12, 22.6%, 22.9%, and 8.2% gained more than 15 letters in the three groups, respectively. The mean central retinal thickness was significantly reduced from baseline with ranibizumab (−119 *μ*m) and ranibizumab plus laser (−128 *μ*m) versus laser (−61 *μ*m; both *p* < 0.001) [23].

In the extension study [24], patients were eligible to receive ranibizumab and concomitant laser treatment. In the prior laser group, a progressive BCVA improvement (+6.0 letters) and CMT reduction (−142.7 *μ*m) at month 36 were observed after allowing treatment with ranibizumab. The prior ranibizumab and ranibizumab plus laser groups improved +8 and +6.7 letters compared to baseline and showed 142 and 146 microns CMT reduction, respectively. Medians of 6 (mean of 6.8) and 4 (mean of 6) injections were performed in the prior ranibizumab and ranibizumab plus laser groups, respectively.

In the RESTORE study, combination therapy achieved similar anatomical outcomes compared to ranibizumab monotherapy but less VA improvement, with a small difference in injection number.

#### 4.2.7. Ranibizumab Monotherapy or Combined with Laser versus Laser Monotherapy in Asian Patients with Diabetic Macular Edema (REVEAL Study)

In this study, 396 Asian diabetic patients were randomized to 0.5 mg ranibizumab, 0.5 mg ranibizumab plus laser, or sham injections plus laser. Greater BCVA improvements were achieved at 12 months in both ranibizumab 0.5 mg groups (+5.9 and +5.7 letters), compared with laser monotherapy (+1.4 letters). Mean CMT reduced significantly from baseline to month 12 with ranibizumab (−134.6 *μ*m) and ranibizumab + laser (−171.8 *μ*m) versus laser (−57.2 *μ*m). A mean of 7.8 and 7 injections was received in the ranibizumab and ranibizumab + laser arms, respectively.

Although not statistically significant, combination therapy achieved better outcomes in terms of anatomical resolution of edema, with slightly less injections needed but similar BCVA change compared to ranibizumab monotherapy.

#### 4.2.8. Ranibizumab 0.5 mg Treat-and-Extend Regimen for Diabetic Macular Edema (RETAIN Study)

Treat-and-extend (T&E) approach progressively increases visits and intravitreal injections intervals when BCVA stability is achieved. This single-masked multicentric study aimed to demonstrate the noninferiority (four-letter margin mean average change in BCVA) of this regimen compared to PRN from baseline to months 1 through 12. Patients were randomized to PRN, T&E, or T&E plus focal/grid laser. The latter group received laser treatment on day 1 followed by retreatment at investigator's discretion, with a 3-month minimum interval recommended between treatments. Both T&E regimens were noninferior to PRN based on mean average BCVA change from baseline to months 1 to 12 (T&E plus laser +5.9 and T&E +6.1 versus PRN +6.2 letters; both *p* < 0.0001). At month 24, no differences were found between either groups, but T&E plus laser and PRN showed slightly better results than T&E alone (mean BCVA +8.3 and +8.1 versus +6.5 letters, resp.). The mean number of injections was similar in both T&E approaches (12.4 and 12.8 in the T&E plus laser and T&E groups) but higher than the PRN group (10.7).

In this study, second-year results showed a tendency toward better visual outcomes with treat-and-extend regimen when associated with laser instead of ranibizumab T&E monotherapy.

#### 4.2.9. Ranibizumab Plus Prompt or Deferred Laser or Triamcinolone Plus Prompt Laser for Diabetic Macular Edema (DRCR.net Protocol I Study)

In 2010, DRCR network published the first results of a trial comparing sham injections plus prompt laser, 0.5 mg ranibizumab plus prompt laser, 0.5 mg ranibizumab plus deferred laser (24 or more weeks), or 4 mg triamcinolone plus prompt laser in 854 study eyes with visual acuity of 20/32 to 20/320. At 1-year follow-up, sham injections plus prompt laser and triamcinolone plus prompt laser achieved the lowest results in terms of mean change in the VA letter score from baseline: +3 and +4, respectively; on the contrary, ranibizumab groups gained a mean of +9 letters in this period of time. In the subset of pseudophakic eyes at baseline, visual acuity improvement in the triamcinolone plus prompt laser group was similar to that in the ranibizumab groups [25]. The expanded 2-year results reported were similar to these results [26].

Sixty-seven percent of the eyes completed the 5-year follow-up. While the ranibizumab arms continued to be treated as the original protocol stablished, the laser alone and triamcinolone plus laser groups were able to be treated with ranibizumab as early as 74 weeks from baseline, for persistent DME with vision impairment. Mean change in ETDRS letter scores from baseline in the four groups was +5, +8, +10, and +7 (original laser, ranibizumab plus laser, ranibizumab plus deferred laser, and triamcinolone plus laser groups, resp.). Original laser group achieved the greatest CMT reduction at 5 years from baseline (−196 microns) compared with ranibizumab and triamcinolone groups (−152, −160, and −40 microns, resp.) [27].

Although ranibizumab plus deferred laser achieved the best VA outcomes, it is noteworthy that ranibizumab plus prompt laser-treated eyes reached similar 5-year VA with lower number of injections needed (median of 17 versus 13, resp.) and a median of 3 focal/grid photocoagulation treatments. Furthermore, the original laser group achieved the best results in terms of CMT reduction.

### 4.3. Clinical Trials with Macular Laser as a Rescue Treatment

#### 4.3.1. Ranibizumab for Diabetic Macular Edema (RISE/RIDE Studies)

Adults with vision loss from DME (BCVA 20/40–20/320 Snellen equivalent) and central subfield thickness of 275 or more were randomized to monthly sham, 0.3 mg or 0.5 mg ranibizumab injections. From month 3, all patients were evaluated monthly for the need for macular laser. Grid or focal photocoagulation directly to microaneurysms with proven leakage in areas of retinal edema was performed if CMT was 250 *μ*m or more; there was less than 50 *μ*m change from the previous visit and the investigator believed it would be of benefit. The mean number of macular laser treatments over 24 months was 1.8 for the sham group and 0.8 in both ranibizumab groups. In RISE, at 24 months, 18% of sham patients gained 15 or more letters versus 45% of 0.3 mg (*p* < 0.0001) and 39% of 0.5 mg (*p* < 0.001) ranibizumab patients. In RIDE, more patients treated with ranibizumab gained 15 letters or more: 12% of sham patients versus 34% of 0.3 mg patients (*p* < 0.0001) and 46% of 0.5 mg ranibizumab patients (*p* < 0.0001). Significant decreases in retinal thickness were achieved, and retinopathy was more stable in the ranibizumab-treated group [28].

In the third year, sham patients, while still masked, were eligible to be treated with monthly 0.5 mg ranibizumab. VA outcomes and reductions in CMT seen at month 24 in the ranibizumab groups remained stable through the last visit. On the other hand, after being treated with ranibizumab, average VA gains in the sham group were lower compared with the gains seen in the ranibizumab patients after 1 year of treatment (+2.8 versus +10.6 and +11.1 letters for prior sham, 0.3 mg and 0.5 mg ranibizumab, resp.) [29].

Once again, laser monotherapy probes to be less effective in improving VA: although 74 and 70% of patients received focal/grid laser during the first 24 months of follow-up in the sham groups, compared to 39 and 36% for 0.3 mg ranibizumab and 35 and 18% for 0.5 mg ranibizumab, final VA in this group was worse than in the ranibizumab groups. The influence of macular laser in number of injections is not evidentially because of the monthly based regimen of the protocol.

#### 4.3.2. Aflibercept, Bevacizumab, or Ranibizumab for Diabetic Macular Edema (DRCR.net Protocol T Study)

Protocol T was the first study designed to compare PRN 2 mg aflibercept, 1.25 mg bevacizumab, and 0.3 mg ranibizumab in center-involved DME in 660 patients.

Although laser treatment was not part of study arms, it was performed after 6 months if DME persisted. Aflibercept group received fewer laser treatments (41%), compared with bevacizumab and ranibizumab groups during the 2 years of follow-up (64% and 52%, resp.) (aflibercept versus bevacizumab, *p* < 0.001; aflibercept versus ranibizumab, *p* = 0.04; bevacizumab versus ranibizumab, *p* = 0.01). Fewer injections were needed during the second year of follow-up in all groups: 5, 6, and 6 compared with 10, 10, and 9 for aflibercept, bevacizumab, and ranibizumab, respectively. Aflibercept-, bevacizumab-, and ranibizumab-treated eyes showed VA improvement from baseline to 2 years, but VA outcomes among the eyes with worse baseline VA were better with aflibercept compared with bevacizumab but not compared with ranibizumab. Similarly, CMT decreased on average by 171, 126, and 149 microns for aflibercept, bevacizumab, and ranibizumab, respectively [30].

Even though aflibercept-treated eyes received less laser treatment, it is not possible to assess the separate effect of macular laser on visual and anatomical outcomes in each treatment arm because it was part of the treatment regimen in this study.

## 5. Conclusions

Since the ETDRS showed that focal/grid laser photocoagulation reduced moderate vision loss from DME by 50% or more, laser became the gold standard for the treatment of DME. Twenty-five years later, intravitreal triamcinolone showed no long-term benefit in VA improvement; thus, laser continued to be the first-line treatment option for DME. However, since the development of anti-VEGF drugs, better VA results were obtained as well as a greater decrease in macular thickness when antiangiogenic drugs alone were compared with laser monotherapy. Currently approved ranibizumab (0.3 mg in the USA and 0.5 mg in Europe) and 2 mg aflibercept, as well as off-label 1.25 mg bevacizumab, have become nowadays the first-line therapy for center-involving DME.

However, macular laser therapy may still have an important role as an adjuvant treatment, as studies with bevacizumab, ranibizumab, or aflibercept have shown that a synergic effect can be achieved when laser is combined with antiangiogenics. Laser effect, despite being slower than antiangiogenics, can have a longer lasting effect and seems to increase over time while injections have more stable long-term results. Furthermore, combination therapy may reduce the chance of secondary engorgement of the laser burns, as the prompt effect of the antiangiogenic drying the macula reduces the intensity of the laser required. The change of macular thickness at the final visit from baseline may be further reduced when laser is added to injections, as described in READ-2 study with 3 years of follow-up; this has been also described in a short-term case series at 12 months (152 versus 143 microns reductions with bevacizumab monotherapy and combination therapy, resp.) [31]. Also, in DRCR.net Protocol I, prior laser monotherapy group with ranibizumab added from week 74, achieved the greatest CMT reduction at 5 years. In the Protocol T, to achieve a totally dry macula, laser needed to be done after six months of intravitreal therapy in 41%, 52%, or 64% of the patients treated with aflibercept, ranibizumab, or bevacizumab, respectively. However, in a meta-analysis of 12–36 months of follow-up studies, no differences were found in this regard between ranibizumab monotherapy and combined with laser [32], and in a multicentric study comparing bevacizumab monotherapy versus combination with laser, bevacizumab showed better functional and anatomic results at 24 months than combination therapy [33].

Additionally, number of needed injections may be reduced by the adjuvant effect of laser treatment, as demonstrated by READ-2, RESTORE, and Protocol I studies. Small case series also found a reduced number of injections needed when combining laser with ranibizumab (mean of 2.4 versus 3.3 injections during a mean of 14 months of follow-up, resp.). This study also found that the mean duration between injections was significantly reduced in the combination therapy [34].

Newer technologies like imaged-guided photocoagulation systems and short-pulse lasers may improve laser outcomes. Combination therapy with bevacizumab injections, followed by navigated laser treatment applied after retinal thinning, required a mean of 4.4 injections during the 12 months of follow-up [35]. Likewise, combination therapy with short-pulse focal/grid photocoagulation required 3.4 ranibizumab injections in six months when no apparent microaneurysms were present, presumably because of a reduced influx of fluid into the treated macula [36].

Thus, although monotherapy macular laser treatment seems to have lost its role as a gold standard treatment for DME involving the center of the macula, it may still play an important role when combined with antiangiogenics helping to reduce macular thickness and number of injections needed.
